# Controlled Growth of LDH Films with Enhanced Photocatalytic Activity in a Mixed Wastewater Treatment

**DOI:** 10.3390/nano9060807

**Published:** 2019-05-28

**Authors:** Zhongchuan Wang, Pengfei Fang, Parveen Kumar, Weiwei Wang, Bo Liu, Jiao Li

**Affiliations:** 1 School of Material Science and Engineering, Shandong University of Technology, Zibo 255000, China; wangzhongchuan1994@163.com (Z.W.); 17864373857@163.com (P.F.); haiyan9943@163.com (J.L.); 2 Laboratory of Functional Molecular and Materials, School of Physics and Optoelectronic Engineering, Shandong University of Technology, Zibo 255000, China; kumar@sdut.edu.cn

**Keywords:** LDHs, film, mixed wastewater, photocatalytic activity

## Abstract

Due to multiple charge transport pathways, adjustable layer spacing, compositional flexibility, low manufacturing cost, and absorption of visible light, layered double hydroxides (LDHs) are a promising material for wastewater treatment. In this study, LDH films and Fe-doped LDH films with different metal ions (Ni, Al, Fe) on the surface of conductive cloth were successfully prepared and applied for the photocatalytic degradation of wastewater containing methyl orange and Ag ions under visible-light irradiation. The chemical state of Fe ions and the composition of LDHs on methyl orange photodegradation were investigated. The experimental results showed that LDH films exhibited high photocatalytic activity. The photocatalytic activity of LDH films on methyl orange improved in the mixed wastewater, and the Fe-doped NiAl–LDH films exhibited best visible-light photocatalytic performance. The analysis showed that Ag ions in the mixed wastewater were reduced by the LDH films and subsequently deposited on the surface of the LDH films. The Ag nanoparticles acted as electron traps and promoted the photocatalytic activity of the LDH films on methyl orange. Thus, we have demonstrated that prepared LDH films can be used in the treatment of mixed wastewater and have broad application prospects in environmental remediation and purification processes.

## 1. Introduction

Heavy metal ions and organic compounds are often discharged together during many industrial processes such as metal finishing, petroleum refining, and leather tanning and finishing, which indicates that the coexistence of heavy metal ions and organic compounds in wastewater is a common phenomenon [1,2,3]. For example, heavy metal ions such as Ag^+^, Cr^6+^, Cu^2+^, generated from rinsing of plated articles, often coexist with organic dyes such as methyl orange. Many studies have focused on single pollutant treatments, however, it is often difficult to treat mixed contaminants using photocatalysts designed for single pollutants [4]. Therefore, the design and preparation of photocatalysts which can be used to treat the mixed contaminants of heavy metal ions and organic compounds are very important for practical pollutant remediation.

Among the developed photocatalytic materials, layered double hydroxides (LDHs) can be considered ideal photocatalysts for the treatment of various types of pollutants due to multiple charge transport pathways, adjustable layer spacing, compositional flexibility, low manufacturing cost and absorption of visible light. Many studies have been reported on the photocatalytic degradation of heavy metal ions, organic compounds, and CO_2_ using LDHs as the photocatalyst [5,6,7,8]. The composition of LDHs with metal ions of variable valences (such as Co, Ni, Fe, Cr) could offer an effective pathway for electron-hole transportation and help to capture heavy metal ions in solution. For example, the Co(II) in CoAl–LDHs helps to capture Pd(II) species through an in situ redox reaction, resulting in the formation of Pd nanoclusters monodispersed on the surface of CoAl–LDHs [9]. It has also been shown that Au/Cr-substituted hydrotalcite could be an efficient heterogeneous catalyst for aerobic alcohol oxidation, owing to the formation of the Cr^3+^–Cr^6+^ redox cycle [10]. In addition, NiFe–LDHs have multiple electronic excitation pathways via metal-to-metal charge transfer through Ni^2+^–O–Fe^3+^, d–d transitions of Ni^2+^, and ligand-to-metal charge transfer through O–Ni^2+^/Fe^3+^ [11].

The effective properties of photocatalytic materials could further be improved by the modification of metal ions or the formation of innovative compositions. For example, Ag@Ag_3_PO_4_/g-C_3_N_4_/NiFe–LDH nanocomposites improved the photocatalytic ability for the reduction of Cr(VI) to Cr(III) [5]. Similarly, Ag nanoparticle-coated Zn/Ti–LDH composites showed higher photocatalytic activity for rhodamine B (RhB) and NO, owing to the formation of Schottky barriers between LDH and Ag nanoparticles and the surface plasmon resonance effect of Ag nanoparticles [12]. Furthermore, NiFe–LDHs modified through ion doping with [M(C_2_O_4_)_3_]^3−^ (M = Cr and Rh) exhibited higher magnetic properties [13]. In another study, Tb^3+^-doped CaAl–LDHs showed an enhancement in fluorescence intensity [14], H_2_SrTa_2_O_7_ with difference photocatalytic activities toward CO and H_2_ evolution was obtained using various metals as cocatalysts (Ag, Pd, Au, and Cu_2_O) [15], and the photocatalytic properties of ZnS were improved by modification with Ru nanoparticles [16]. Similar studies have shown that the formation of dual surface heterostructure by deposing Au and CuO on Cu_2_O cubes improved the photocatalytic activity of Cu_2_O due to the synergistic effect of CuO/Cu_2_O and Au/Cu_2_O [17]. However, in one study, the diffusion of K^+^ ions from poly(heptazine imide) (PHIK) to a metal–organic framework (MOF) made PHIK more negatively charged and MOF more positively charged, which provided the strongest interaction between PHIK and MOF and resulted in superior photocatalytic activity through rhodamine B degradation [18]. It has also been shown that the formation of heterojunctions could increase photocatalytic properties, such as Cu_2_S/ZnO, Cu_2_O/TiO_2_, and CuInS_2_/TiO_2_ [19,20,21], and that the presence of Na_2_S hole scavengers increased the photoreduction of CO_2_ to form (HCOO^−^) on ZnS [22]. Finally, due to the different Fermi levels between heavy metals and LDHs, heavy metals deposited on the surface of LDHs could act as electron traps to prevent electron-hole recombination [5,9,10], which would improve the photocatalytic performance of LDHs.

According to the above discussion, LDHs could be used for the photocatalytic degradation of mixed wastewater. However, LDH powders exhibit the following drawbacks: low photocatalytic activity, formation of aggregates, and difficulties in subsequent separation processes. Using a two-dimensional structure with excellent carrier mobility as the substrate is an effective approach for enhancing the photocatalytic efficiency by preventing agglomeration and decreasing charge recombination [23,24]; besides, the formation of films on the substrate is beneficial for recovering the catalysts from the solution. Among those substrates, conductive cloth has the characteristics of good conductivity, flexibility, and low manufacturing cost. From this viewpoint, we used conductive cloth as the substrate and LDHs as the active sites for photocatalytic domains. Based on the photo-induced reduction and photocatalytic degradation of LDHs, LDH films were successfully used to treat the mixed wastewater containing heavy metal ions and organic compounds. The formation of heavy metals and LDHs on the surface of the conductive cloth led to an enhancement of the photocatalytic activity for organic pollutants degradation and, thereby, realized the photocatalytic treatment of mixed wastewater.

## 2. Materials and Methods

### 2.1. Materials

Al(NO_3_)_3_·9H_2_O, Ni(NO_3_)_2_·6H_2_O, Fe(NO_3_)_3_·9H_2_O, AgNO_3_, ferric ammonium citrate ((NH_4_)_3_·[Fe(Cit)_2_]), urea, methyl orange, and ammonium fluoride (NH_4_F) were purchased from Sinopharm Chemical Reagent Co. Ltd. (Shanghai, China) and used as received without further purification. The conductive cloth was purchased from Zhongyang shielding material production company, Guangzhou, China.

### 2.2. Synthesis of LDH Films

NiAl–LDH films were prepared using the method previously reported with some minor modifications [25]. The conductive cloth (2 cm × 6 cm) was cleaned with a mixed solution of deionized water, ethanol, and acetone (volume ratio, 1:1:1) in an ultrasonic bath for 30 min. Ni(NO_3_)_2_·6H_2_O (0.15 mol·L^−1^), Al(NO_3_)_3_·9H_2_O (0.05 mol·L^−1^), NH_4_F (0.2 mol·L^−1^), and urea (0.5 mol·L^−1^) were dissolved in deionized water under magnetic stirring to form a clear solution at room temperature. The conductive cloth was vertically placed in the solution without stirring and heated at 110 °C for 8 h to form a thin film on its bottom side, which was washed by deionized water. For NiFe–LDH films, Fe(NO_3_)_3_·9H_2_O (0.05 mol·L^−1^) was used instead of Al(NO_3_)_3_·9H_2_O. LDH powders were also prepared under the same experimental conditions as that of LDH films but without conductive cloth.

Fe-doped LDH films: LDH films were vertically placed in a (NH_4_)_3_·[Fe(Cit)_2_] solution (0.01 mol·L^−1^) and heated at 75 °C for 12 h to form Fe-doped LDH films, which were cleaned with deionized water. For Fe-doped LDH powders, LDH powders were used instead of LDH films.

### 2.3. Characterization

X-ray powder diffraction (XRD) patterns were recorded using a D8 ADVANCE X-ray diffractometer (Karlsruhe, Germany) with Cu K_α_ radiation (λ = 0.15406 nm). The scanning electron microscopy (SEM) images were recorded on a FEI-Sirion 200 F field emission scanning electron microscope (Hongkong, China). The transmission electron microscopy (TEM) images, high-resolution transmission electron microscopy (HRTEM) images, and the energy dispersive spectroscopy (EDS) spectra were taken with a FEI-Tecnai G2 field emission transmission electron microscope (Hongkong, China). The samples were obtained by peeling off LDH films from the substrate. Photocatalytic reactions were carried out using a 300 W Xe lamp as the light source and the light intensity in the visible region was about 85 mW·cm^−2^. The UV–Vis diffuse reflectance spectra were recorded by a UV–Vis spectrophotometer (UV-3900H, Hitachi, Tokyo, Japan). Fourier transform infrared (FTIR) spectra were recorded by a Thermo Nicolet 5700 (Waltham, MA, USA). The chemical state of LDHs was investigated by X-ray photoelectron spectroscopy (XPS) on a ESCALAB 250Xi photoelectron spectrometer (Waltham, MA, USA) with Al K (1486 Ev) as the excitation light source. The N_2_ adsorption/desorption tests were measured by Brunauer–Emmett–Teller (BET) measurements using a NOVA2200e surface area analyzer (Boynton Beach, FL, USA).

### 2.4. Photocatalytic Property Measurement

LDH films (2 cm × 6 cm) were immersed in a mixed solutions of methyl orange (20 mg·L^−1^) and AgNO_3_ (0, 5, 10, 20, and 30 mg·L^−1^) and kept in the dark for 30 min to ensure adsorption–desorption equilibrium. The pH value of the solution was about 6. Samples were removed from the solution after various irradiation times and analyzed using a UV–Vis spectrophotometer at 464 nm. All photocatalytic experiments were repeated three times. The weight of the photocatalyst was calculated from the formula *m = m*_1_ − *m*_0_, where *m*_0_ and *m*_1_ represent the weight of the substrate before and after LDH growth, respectively. The LDHs grown on conductive cloth were about 6.6, 6.9, 6.5, and 6.7 mg for NiAl–LDH films, NiFe–LDH films, Fe-doped NiAl–LDH films, and Fe-doped NiFe–LDH films, respectively. To test the photocatalytic performance of LDH powders, LDH powders (15 mg) were used instead of LDH films.

## 3. Results and Discussion

XRD analysis was performed to confirm the crystal structure and phase (Figure S1). Both the structure of NiAl–LDH films and NiFe–LDH films matched well to the typical LDH lamellar structure (JCPDS File No. 48-0594 and 51-0463). According to the (003), (006), and (009) reflections, the basal spacing values were 0.756 nm (NiAl–LDHs) and 0.764 nm (NiFe–LDHs), which coincide well with the values for CO_3_^2−^-intercalated LDH materials [26]. The Fe-doped LDH films displayed a phase of lamellar structures with a slightly lower degree of crystallinity and basal spacing (0.755 and 0.762 nm for Fe-doped NiAl–LDH films and Fe-doped NiFe–LDH films, respectively) which was lower than that of Fe complex-intercalated LDHs (1.214 nm) [27]. This suggests that the interlayer ion of LDHs was CO_3_^2−^ and that no Fe complex intercalated in the interlayer. According to the (003) crystal plane spacing, grain size and stacking numbers of LDHs in the direction of the *c* axis were calculated using the Scherrer formula. The grain size for NiFe–LDH films and NiAl–LDH films were 43.2 and 33.9 nm, respectively, whereas the stacking numbers for NiFe–LDH films and NiAl–LDH films were 57 and 45, respectively.

**Figure 1:**
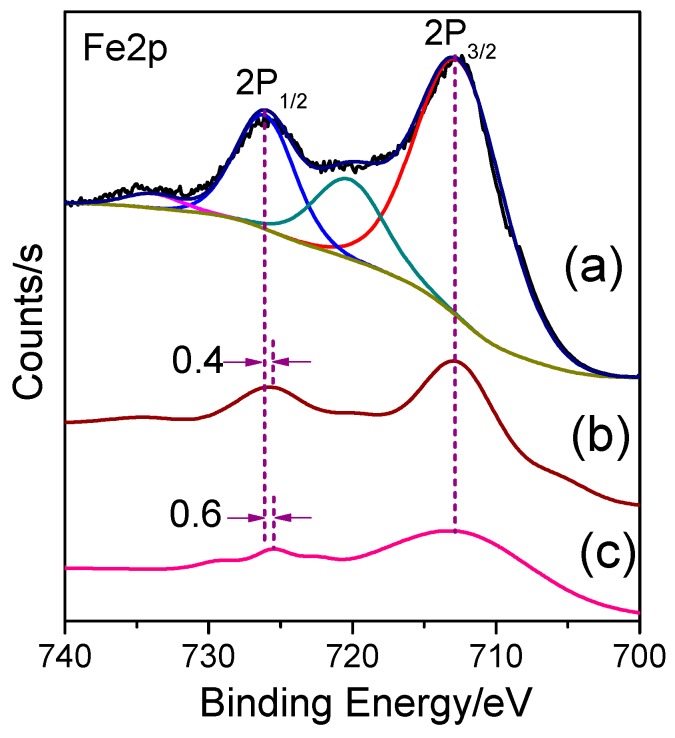
The high-resolution Fe 2p XPS spectra of (**a**) NiFe–LDH films, (**b**) Fe-doped NiFe–LDH films, and (**c**) Fe-doped NiAl–LDH films.

The chemical state of Fe ions in the LDH films was investigated by XPS spectra. The peaks at 726.28 and 712.78 eV are attributed to the 2p1/2 and 2p3/2 spin states of Fe(III) for LDH lamellar structure [28,29,30,31,32] as seen in Figure 1a. After Fe ion doping, the positions of Fe 2p3/2 were basically the same (within the experimental uncertainty [33]), while the peaks for Fe 2p1/2 in both LDH films showed a negative shift of ~0.4 and 0.6 eV, as shown in Figure 1b,c. The mole ratio of Fe/Ni for Fe-doped NiFe–LDH films and Fe-doped NiAl–LDH films were 0.58:1 and 0.38:1, respectively. The increase in the mole ratio of Fe/Ni after doping indicates successful doping of Fe ions. No peaks for Fe complexes (725.0 and 711.5 eV) were observed, confirming that no Fe complex intercalated into the LDH films [28].

**Figure 2:**
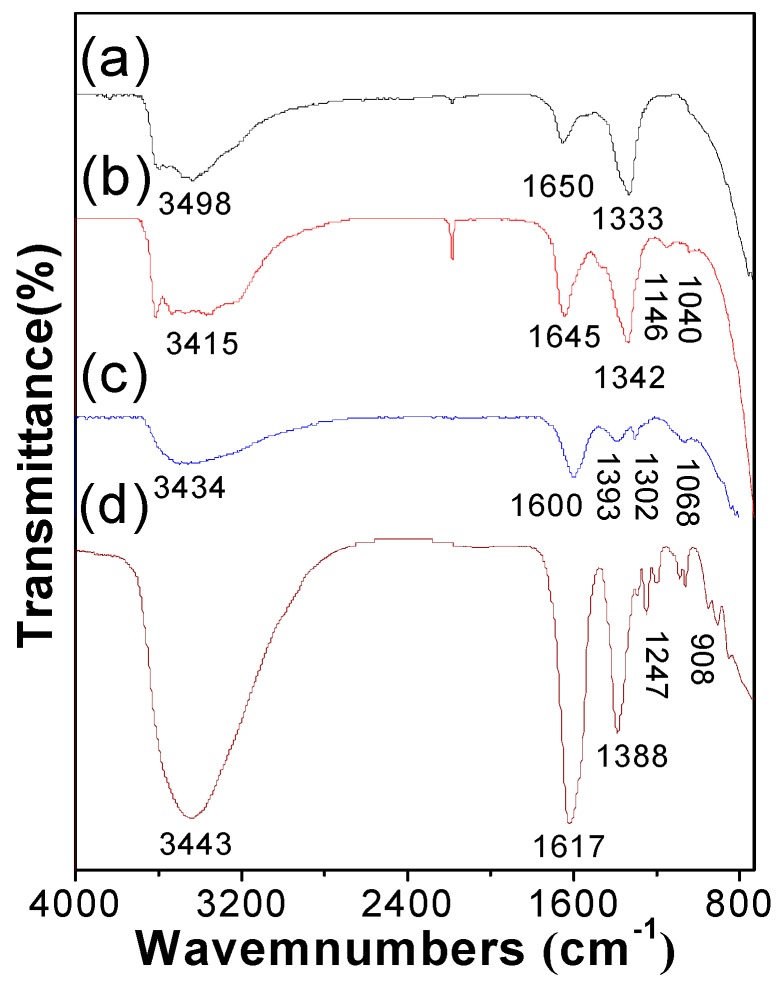
FTIR spectra of (**a**) NiFe–LDH films, (**b**) NiAl–LDH films, (**c**) Fe-doped NiAl–LDH films, and (**d**) Fe-doped NiFe–LDH films.

As seen in Figure 2, FTIR spectra of all LDH films provide evidence for the presence of intercalated CO_3_^2−^ and interlayered water. After Fe(III) doping, the peak position of CO_3_^2−^ moved toward high frequency (around 1388 cm^−1^) due to the change in charge density by doping Fe(III) into the LDH films [34]. No vibration peak (1290 cm^−1^) for an Fe complex appeared.

**Figure 3:**
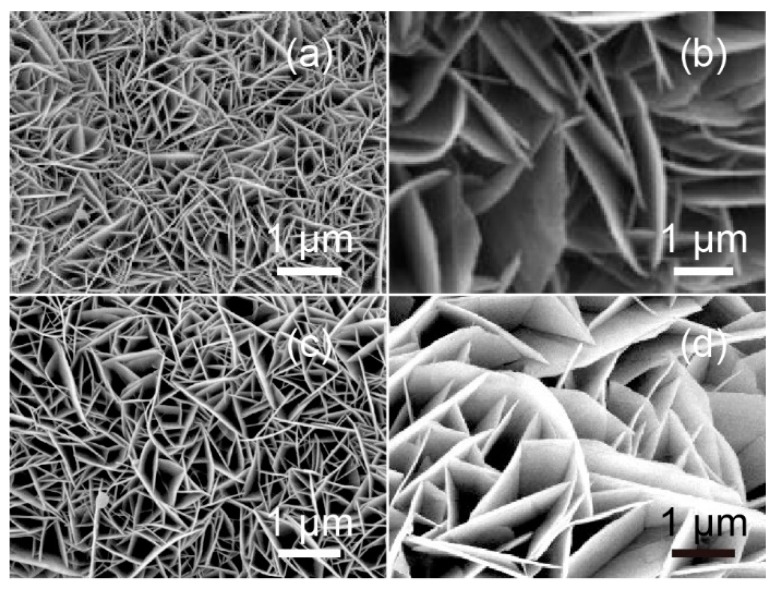
SEM images of (**a**) NiAl–LDH films, (**b**) NiFe–LDH films, (**c**) Fe-doped NiAl–LDH films, and (**d**) Fe-doped NiFe–LDH films.

**Figure 4:**
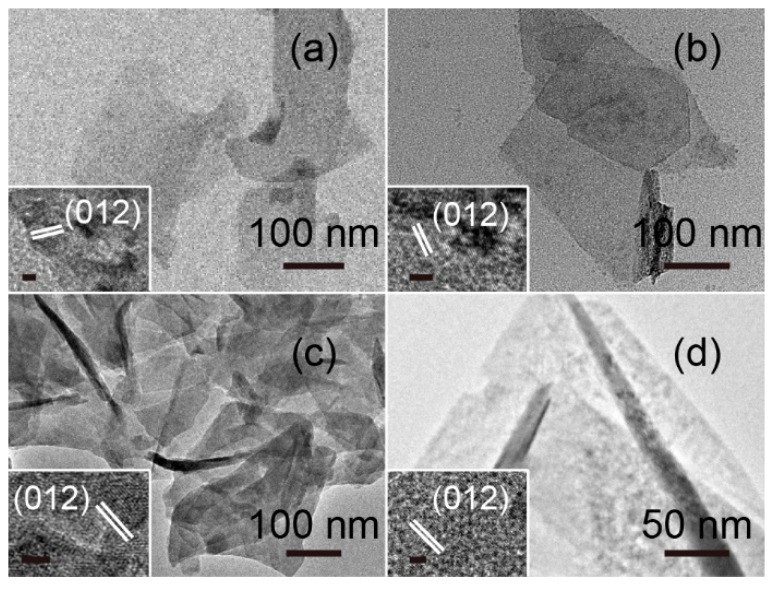
TEM images of (**a**) NiAl–LDH films, (**b**) NiFe–LDH films, (**c**) Fe-doped NiAl–LDH films, and (**d**) Fe-doped NiFe–LDH films. Insets are corresponding HRTEMs, scale bar: 2 nm.

Figure 3 shows morphology images of the LDH films obtained using SEM analysis and suggests typical sheet-like structures uniformly distributed on the surface of the substrate, thus effectively solving the problem of powder agglomeration. Figure 3a shows NiAl–LDH sheets intersecting and aligned vertically on the conductive cloth under the orienting function of NH_4_F [10]. NiFe–LDH films also exhibited a similar sheet-like morphology, as seen in Figure 3b, but the sheets were larger and more loosely distributed. The composition of the LDHs affected the sheet thickness, which was consistent with the results from XRD analysis. After Fe ion doping, NiAl–LDH and NiFe–LDH sheets displayed a lamellar structure of similar size. TEM investigation of LDH films revealed the almost transparent, thin, layered morphology of the sheets, as shown in Figure 4. All HRTEM images exhibited clear lattice fringes with a d-spacing value of 0.2593 nm corresponding to (012) reflection from the LDHs.

**Figure 5:**
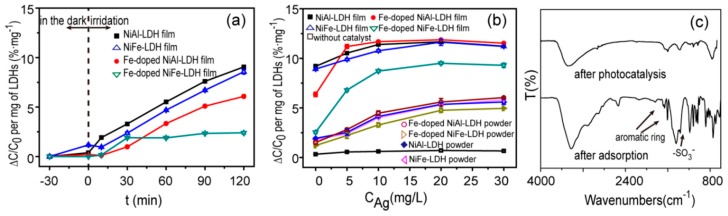
(**a**) Photocatalytic activity of methyl orange for LDH films without Ag ions. (**b**) Effect of Ag ion concentration on degradation of methyl orange under 120 min illumination. C_0_: the equilibrium concentration of methyl orange before irradiation, ∆C: the change in the concentration of methyl orange after irradiation. (**c**) FTIR spectra of NiAl–LDH films after photocatalytic experiments and adsorption experiments.

The photocatalytic activity in methyl orange degradation under visible-light irradiation was evaluated, as shown in Figure 5a. All LDH films showed a weak adsorption effect after treatment in a dark place for 30 min. Figure 5b illustrates the blank experiment—in the absence of catalysts but under irradiation, showed that a small quantity of methyl orange was degraded Figure 5b. No obvious photosensitive degradation of methyl orange was observed. The degradation of methyl orange mainly depends on photocatalytic activity, which tends to increase with increasing irradiation time. In general, the photocatalytic activity depends on the amount of catalyst. Within a certain range, the use of more photocatalyst provides higher photocatalytic activity [35]. In our experiments, the degradation of methyl orange per milligram of LDHs was used to describe the photocatalytic activity. Under 120 min illumination, the degradation of methyl orange for NiAl–LDH and NiFe–LDH films was 9.1%·mg^−1^ and 8.4%·mg^−1^, respectively. When Fe-doped LDH films were used as a photocatalyst, the degradation of methyl orange decreased.

**Figure 6:**
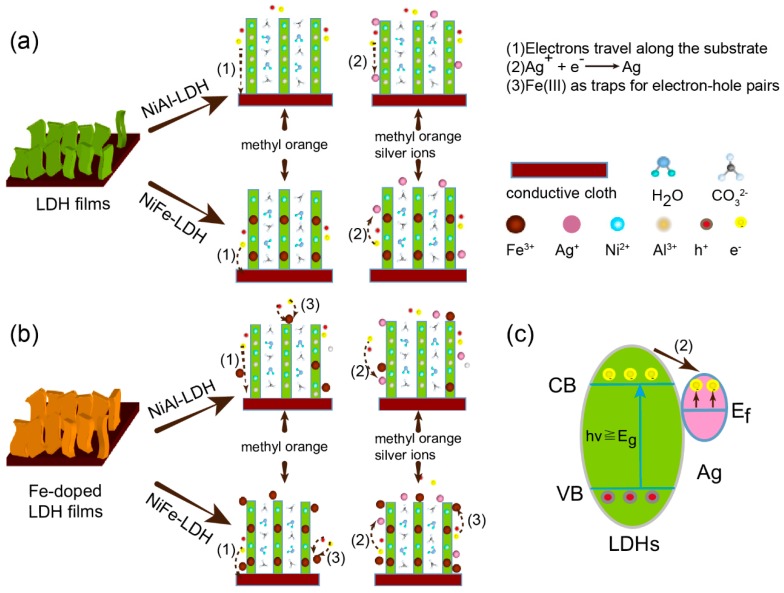
Schematic diagram of possible electron transport in (**a**) LDH films and (**b**) Fe-doped LDH films in degradation of methyl orange or a mixed solution of methyl orange and Ag ions. (**c**) Possible electron transport between LDHs and Ag nanoparticles.

The Fe(III) ionic radius (64.5 pm) is larger than Al(III) ionic radius (53.5 pm); hence, the replacement of Al(III) by Fe(III) could increase the distance between metal ions in the layer and reduce the charge density. This is unfavorable to electron-hole transfer throughout the layered framework, and thereby leads to a low level of photocatalytic activity as shown in Figure 6a [5,7]. Therefore, NiAl–LDH films showed better photocatalytic activity compared to NiFe–LDH films. The Figure 6b pathway 3 shows that after Fe doping, Fe(III) ions act as traps for photogenerated electron-hole pairs and accelerate electron-hole pair recombination [36,37,38], thus decreasing the photocatalytic degradation of methyl orange by Fe-doped LDH films.

The photocatalytic activity of LDH films was studied in mixed wastewater containing Ag ions and methyl orange. The degradation of methyl orange was gradually accelerated when the concentration of Ag ions increased from 0 to 20 mg·L^−1^, as seen in Figure 5b. Fe-doped NiAl–LDH films exhibited the best photocatalytic activity with 20 mg·L^−1^ Ag ion concentration and 11.92%·mg^−^^1^ degradation rate of methyl orange, which is higher than reported values (degradation of methyl orange for xanthan gum/TiO_2_ [35], MnO_2_-M [39], and WO_3_/g-C_3_N_4_ [40] were 2.3%, 9.5%, and 4.84%·mg^−^^1^, respectively). When the concentration of Ag ions in solution was further increased from 20 mg·L^−1^, the degradation of methyl orange decreased slightly due to the light shielding effect of Ag ions adsorbed on the surface of LDHs.

In the presence of Ag ions, the degradation of methyl orange by LDH films was higher as seen in Figure 5b. Figure 6, pathway 2 shows Ag nanoparticles were reduced from the solution and deposited on the surface of the LDH films, which received electrons and promoted the separation of photogenic electron-hole pairs [41], thus improving the photocatalytic activity of LDH films. That is, the electron traps of Ag nanoparticles prevented the recombination of electron-hole pairs on Fe(III) ions. The capture of electrons and holes by Ag nanoparticles and Fe(III) ions promoted the separation of electron-hole pairs. As a result, when the concentration of Ag ions increased from 0 to 20 mg·L^−1^, there was a large increase in the degradation of methyl orange by Fe-doped LDH films, as shown in Figure 5b.

For comparison, corresponding LDH powders were also prepared. All of the LDH powders showed similar sheet-like morphology and lamellar structure to that of LDH films, as shown in Figures S2 and S3. However, as seen in Figure 5b, the photocatalytic degradation of methyl orange by LDH powders was lower than that of LDH films, which confirms that the conductive cloth could facilitate the transportation of photogenerated charges shown in Figure 6a,b, pathway 1 [23]. However, this comparison is qualitative and we will further study the kinetics of excitons and free carriers in LDH films, for example, the charge transfer dynamics, to obtain the quantitative properties of LDH films [42,43,44]. In addition, LDH films could be easily separated from the solution, providing a simple method for the recovery of the catalyst.

We also used FTIR spectra to investigate LDH films after photocatalytic degradation and adsorption experiments, as shown in Figure 5c. After photocatalytic degradation of methyl orange, the peak intensity of CO_3_^2−^ decreased and no obvious peaks for methyl orange were observed. While after adsorption experiments, some peaks for methyl orange were observed. The differences between photocatalytic degradation and adsorption experiments confirm that the degradation of methyl orange in the presence of LDH films was not through adsorption.

**Figure 7:**
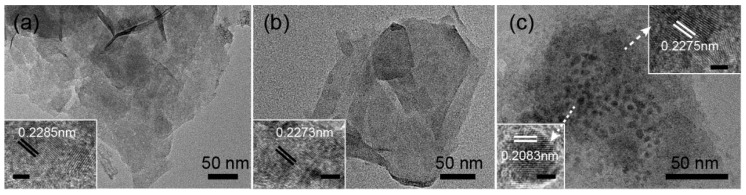
TEM images after photocatalytic reaction (**a**) NiAl–LDH films, (**b**) Fe-doped NiAl–LDH films, and (**c**) Fe-doped NiAl–LDH films in the presence of 5 mg·L^−1^ Ag ions. Insets are corresponding HRTEM images. Scales in insets are 2 nm.

The LDH films after the photocatalytic reaction were examined by TEM and EDS. No noticeable change was observed in the morphology of LDH films before and after the photocatalytic reaction. LDH films exhibited good stability for the degradation of methyl orange. As shown in Figure 7, the LDH sheets were transparent to the electron beam, suggesting that they were very thin. The insets in Figure 7a–c show that after the photocatalytic reaction, the HRTEM images taken from one sheet can be indexed to the (*0 0 10*) plane, which matches well with the reported values of the LDH structure. Figure 7c shows that in the presence of Ag ions, some Ag nanoparticles were observed on the surface of LDH sheets. The lattice spacing of 0.2083 nm can be indexed to the (*200*) plane of Ag nanoparticles. EDS spectra also confirmed the formation of Ag after photocatalytic reaction, as seen in Figure S5.

## 4. Conclusions

LDH films and Fe-doped LDH films with different compositions (Ni, Al, and Fe) on the surface of conductive cloth were successfully prepared and applied for photocatalytic degradation of methyl orange in mixed wastewater. All LDH films showed layered structures and were distributed uniformly on the surface of the substrate. They exhibited high photocatalytic performance and could be easily separated from the solution, providing a simple method for the recovery of the catalyst. Benefiting from the electron trap of Ag nanoparticles, the photocatalytic activity of LDH films on methyl orange was improved, and the Fe-doped NiAl–LDH films presented the best visible-light photocatalytic performance. Our study indicates that LDH films can be used in the treatment of mixed wastewater and have broad application prospects for environmental remediation and purification processes. In order to better realize the application of LDH films in wastewater treatment, further research is needed, including 1) to explore different conductive substrates with excellent carrier mobility which could influence the photocatalytic performance of LDHs; and 2) to explore the reducing capacity of LDH films to other heavy metal ions with different Fermi levels in order to prevent hole-electron recombination, thereby enhancing photocatalytic activity. Based on such research, the factors affecting the photocatalytic performance of LDH films can be better understood. By adjusting the structure and composition of LDHs, different types of mixed wastewater containing heavy metal ions and organic compounds can be selectively treated. We envision that our findings will help in further development of new, improved, and more effective LDH films with enhanced photocatalytic activity for the treatment of mixed wastewater.

## Supplementary Materials

The following are available online at https://www.mdpi.com/2079-4991/9/6/807/s1, Table S1: XPS Peak positions for Fe^3+^ obtained from LDH films and Fe-doped LDH films. Figure S1: XRD patterns. (a) NiAl–LDH films, (b) NiFe–LDH films, (c) Fe-doped NiAl–LDH films, and (d) Fe-doped NiFe–LDH films. Figure S2: SEM images. (a) NiAl–LDH powders, (b) NiFe–LDH powders, (c) Fe-doped NiAl–LDH powders, and (d) Fe-doped NiFe–LDH powders. Figure S3: XRD patterns. (a) NiAl–LDH powders, (b) Fe-doped NiAl–LDH powders, (c) NiFe–LDH powders, and (d) Fe-doped NiFe–LDH powders. Figure S4: EDS elements mapping for Fe-doped NiAl–LDH films after the photocatalytic degradation in the presence of methyl orange (20 mg·L^−1^) and Ag ions (5 mg·L^−1^). (a) Area without Ag particles, (b) area with Ag particles. Figure S5: EDS spectra for Fe-doped NiAl–LDH films after photocatalytic reaction in the presence of 5 mg·L^−1^ Ag ions. (a) Area with Ag nanoparticles, (b) area without Ag nanoparticles in Figure 7c. Figure S6: N_2_ adsorption/desorption isotherms of (a) NiAl–LDH powders and Fe-doped NiAl–LDH powders, (b) NiFe–LDH powders and Fe-doped NiFe–LDH powders.
