# Supplementary material for: Controlled Growth of LDH Films with Enhanced Photocatalytic Activity in a Mixed Wastewater Treatment
Source: Nanomaterials (Basel). 2019 May 28;9(6):807. doi: 10.3390/nano9060807 (PMC6631146; doi:10.3390/nano9060807)
Supplement: Supplementary file 1 [file nanomaterials-09-00807-s001.pdf]

Supplementary material

Controlled growth of LDH films with enhanced photocatalytic activity in a mixed wastewater treatment

Zhongchuan Wang[1], Pengfei Fang[1], Parveen Kumar[2],Weiwei Wang*[1], Bo Liu*[1,2], and Jiao Li[1]

[1]School of Material Science and Engineering, Shandong University of Technology, Zibo 255000, China; [2]Laboratory of Functional Molecular and Materials, School of Physics and Optoelectronic Engineering, Shandong University of Technology, Zibo 255000, China; wangzhongchuan1994@163.com (Z.W.); 17864373857@163.com (P.F.); kumar@sdut.edu.cn (P.K.); haiyan9943@163.com (J.L.)

*Correspondence: wangweiwei@sdut.edu.cn (W.W.); liub@sdut.edu.cn (B.L.); Tel.: +8615689078202(W.W.); +86-533-2783909 (B.L.)

Table S1. XPS Peak positions for Fe^{3+} obtained from LDH films and Fe-doped LDH films.

Film No.	Peak position (eV)	
	2p1/2	2p3/2
NiAl-LDH films	--	--
NiFe-LDH films	726.28	712.78
Fe-doped NiAl-LDH films	725.88	712.88
Fe-doped NiFe-LDH films	725.68	712.78

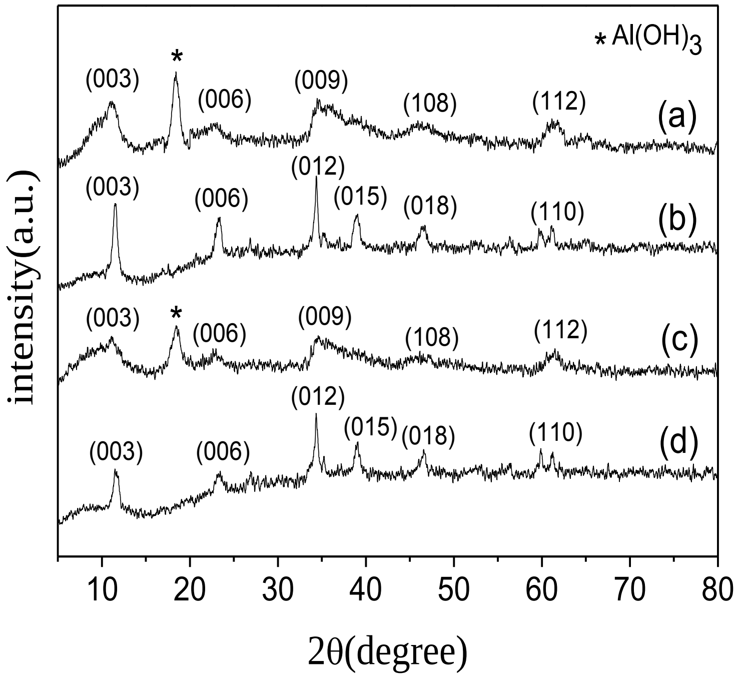

Figure S1. XRD patterns. (a) NiAl-LDH films, (b) NiFe-LDH films, (c) Fe-doped NiAl-LDH films, and (d) Fe-doped NiFe-LDH films.

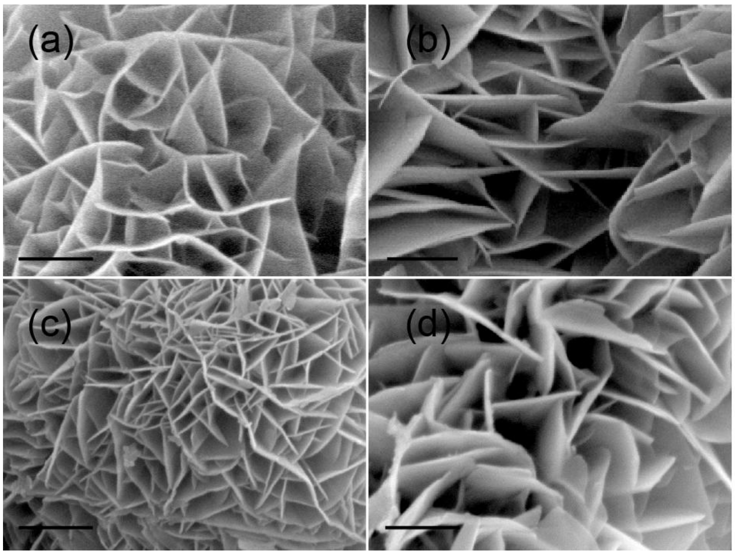

Figure S2. SEM images. (**a**) NiAl-LDH powders, (**b**) NiFe-LDH powders, (**c**) Fe-doped NiAl-LDH powders, and (**d**) Fe-doped NiFe-LDH powders.

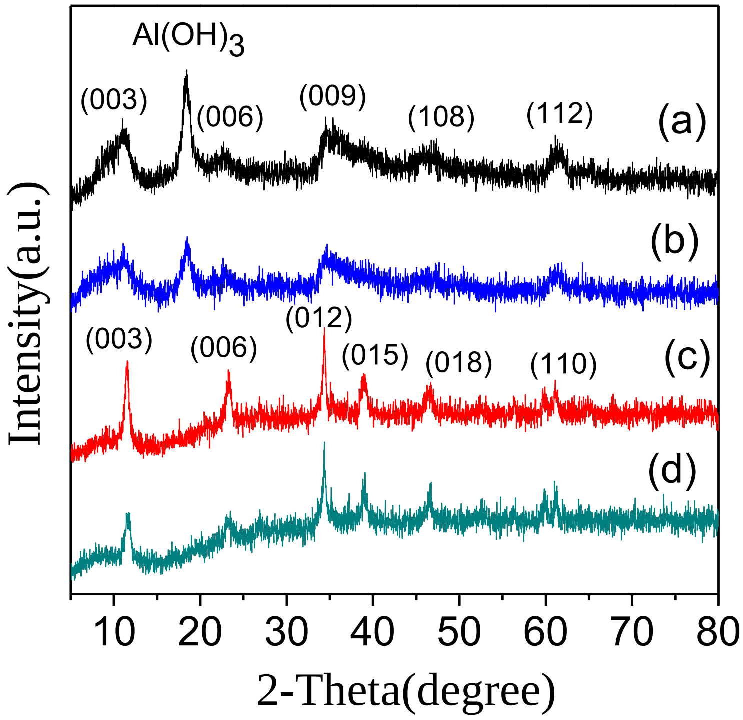

Figure S3. XRD patterns. (**a**) NiAl-LDH powders, (**b**) Fe-doped NiAl-LDH powders, (**c**) NiFe-LDH powders, and (**d**) Fe-doped NiFe-LDH powders.

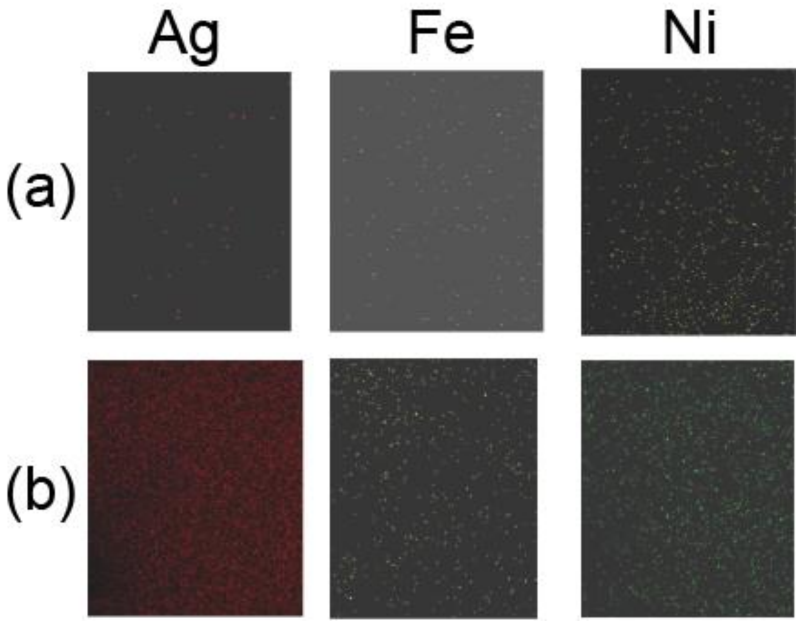

Figure S4. EDS elements mapping for Fe-doped NiAl-LDH films after the photocatalytic degradation in the presence of methyl orange (20 mg·L^{-1}) and Ag ions (5 mg·L^{-1}). (**a**) Area without Ag particles, (**b**) area with Ag particles.

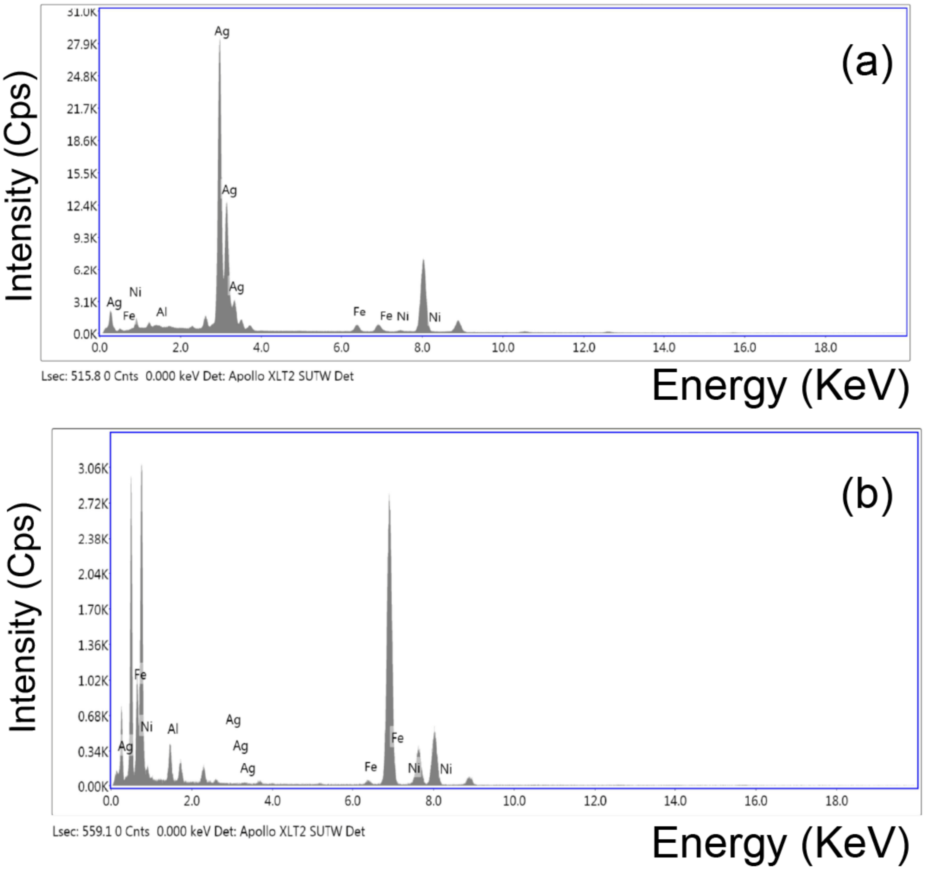

Figure S5. EDS spectra for Fe-doped NiAl-LDH films after photocatalytic reaction in the presence of 5 mg·L^{-1} Ag ions. (**a**) Area with Ag nanoparticles, (**b**) area without Ag nanoparticles in Figure 7(c).

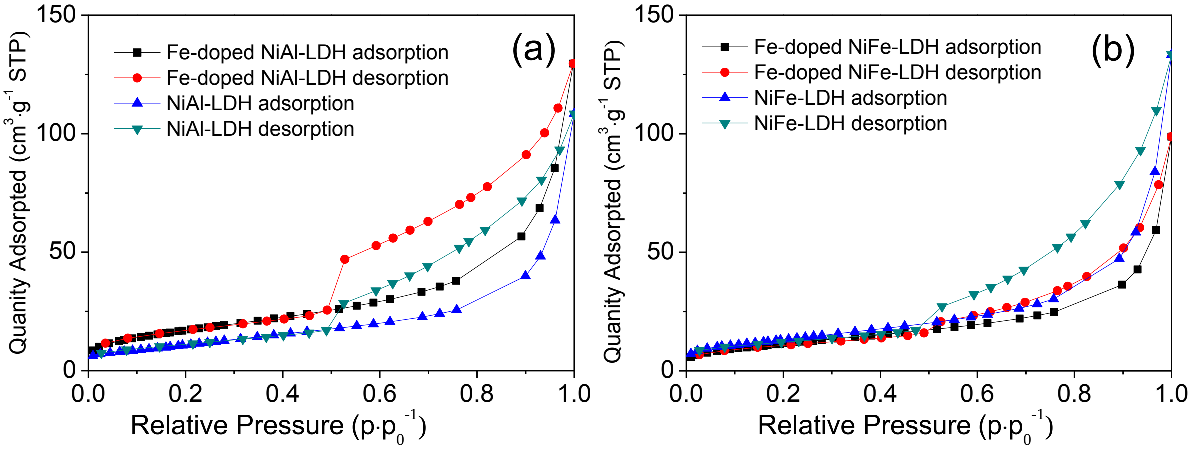

Figure S6. N_2 adsorption/desorption isotherms of (**a**) NiAl-LDH powders and Fe-doped NiAl-LDH powders, (**b**) NiFe-LDH powders and Fe-doped NiFe-LDH powders.

All LDH powders are a type IV isotherm with an H3 hysteresis loop, owing to the aggregation of LDHs nanosheets. The specific surface area for NiAl-LDH powders, NiFe-LDH powders, Fe-doped NiAl-LDH powders, and Fe-doped NiFe-LDH powders are 42.26 $m^2 \cdot g^{-1}$, 43.09 $m^2 \cdot g^{-1}$, 48.37 $m^2 \cdot g^{-1}$, and 42.82$m^2 \cdot g^{-1}$, respectively. The pore size for NiAl-LDH powders, NiFe-LDH powders, Fe-doped NiAl-LDH powders, and Fe-doped NiFe-LDH powders are 14.35 nm, 12.92 nm, 10.38 nm, and 14.27 nm, respectively.
